# Inhibition and assessment of the biophysical gating properties of GluA2 and GluA2/A3 AMPA receptors using curcumin derivatives

**DOI:** 10.1371/journal.pone.0221132

**Published:** 2019-08-27

**Authors:** Mohammad Qneibi, Othman Hamed, Abdel-Razzak Natsheh, Oswa Fares, Nidal Jaradat, Nour Emwas, Qais AbuHasan, Rana Al-Kerm, Rola Al-Kerm

**Affiliations:** 1 Department of Biomedical Sciences, Faculty of Medicine and Health Sciences, An-Najah National University, Nablus, Palestine; 2 Department of Chemistry, Faculty of Science, An-Najah National University, Nablus, Palestine; 3 Department of Computer Information Systems, Faculty of Engineering and Information Technology, An-Najah National University, Nablus, Palestine; 4 Department of Pharmacy, Faculty of Medicine and Health Sciences, An-Najah National University, Nablus, Palestine; University of North Dakota, UNITED STATES

## Abstract

The development of efficacious and safe drugs for the treatment of neurological diseases related to glutamate toxicity has been a focus in neuropharmacological research. Specifically, discovering antagonists to modulate the activity and kinetics of AMPA receptors, which are the fastest ligand-gated ion channels involved in excitatory neurotransmission in response to glutamate. Thus, the current study investigated novel curcumin derivatives on the biophysical properties of AMPA receptors, specifically on the homomeric GluA2 and the heteromeric GluA2/A3 subunits and assessed for inhibitory actions. The biophysical parameter (i.e., desensitization, deactivation, and peak currents) were measured by using whole-cell patch clamp electrophysiology with and without the administration of the derivatives onto HEK293 cells. CR-NN, CR-NNPh, CR-MeNH, and CR-NO of the tested derivatives showed inhibition on all AMPA receptors up to 6 folds. Moreover, the inhibitory derivatives also increased desensitization and deactivation, which further intensifies the compounds’ neuroprotective effects. However, CR-PhCl, CR-PhF, and CR-PhBr did not show any significant changes on the peak current, deactivation or desensitization rates. By comparison to other discovered and widely used antagonist, the prepared curcumin derivatives are not selective to a specific AMPA subunit, instead implement its effect in the same way between all types of AMPA receptors. Additionally, the obtained results provide derivatives that not only noncompetitively inhibit AMPARs but also decrease its biophysical kinetics, specifically desensitization and deactivation rates. Hence, to potentially serve as a new AMPAR inhibitor with therapeutic potential, the current study provides compounds that are non-selective and non-competitive antagonist, which also effect the desensitization and deactivation rates of the receptor.

## Introduction

The amino acid (S)-Glutamate (Glu) is the major excitatory neurotransmitter in the vertebrate central nervous system (CNS). Glutamate targets four types of receptors, three of which are classified as ionotropic glutamate receptors (iGluRs), while the last type of receptors, known as G-coupled proteins, are categorized into the metabotropic receptors family (mGluRs) [1]. The three receptors that the iGluRs consist of have been pharmacologically classified according to the ligands that selectively activate them. Hence, they are regarded as α-amino-3-hydroxy-5-methyl-4-isoxazolepropionic acid receptor (AMPAR), N-methyl-D-aspartate receptor (NMDAR) and kainate receptor, according to their agonists AMPA, NMDA, and kainate respectively. Although all of the iGluRs respond primarily to glutamate and are related in an amino sequence, they have distinct functions in the CNS [1, 2].

All iGluRs function, at a varying degree, in fast synaptic neurotransmission, which is involved in the determination and maintenance of synaptic plasticity that is critical for memory and learning. However, unlike NMDARs, which require both the depolarization of the cell and an agonist stimulation for the opening and activation of the channels, AMPARs are activated by the binding of an agonist to any of the four pore-forming subunits [2, 3]. Hence, AMPARs mediate most of the fast-excitatory neurotransmission as they are activated in a microsecond domain time scale. Nevertheless, it undergoes profound desensitization on the millisecond timescale in favor of a more stable structure [4]. However, excessive activity and fast desensitization and deactivation rates of AMPARs have been linked to neurotoxicity and hypoxic/ischemic insults, which is associated with the pathogenesis of several neurodegenerative and neuropsychiatric diseases, such as Alzheimer Diseases (AD), Parkinson Disease (PD) Epilepsy, Amyotrophic Lateral Sclerosis (ALS) and strokes [5]. It was demonstrated that neuronal death can be triggered from excessive ionic influx via AMPARs, which is linked to numerous neurodegenerative diseases [6–8], for example, the excessive influx of calcium has been correlated with the neurodegeneration of motor neurons in ALS.

Pharmacological treatments, through the synthesis of drugs that act as antagonists of AMPARs to inhibit their activity, have long been the first line in therapy. Structurally different classes of competitive AMPA antagonists have been discovered and heavily studied, such as the quinoxalinediones, isatin oximes, decahydroisoquinoline, and isoxazole derivatives, which bind to the glutamate site of the receptor [9]. On the contrary, non-competitive AMPA antagonists, such as phthalazine derivatives, 3-aryl-quinazoline-4one derivatives, and phenyl1,2,4oxadiazolyl-phenoxy-ethylamine bind to an allosteric site. As a result noncompetitive antagonist are of greater importance from a therapeutic point of view since they are effective even at extremely high concentrations of glutamate [10]. However, many of the researched drugs fail at the clinical trials due to the complexity of drug synthesis and/or low efficacy [11]. Thus, we synthesized novel compounds from curcumin to potentially serve as new AMPAR inhibitors with therapeutic potential and to better understand the biophysical gating properties of these receptors.

The natural polyphenol curcumin possesses many protective properties that are beneficial for the treatment of many diseases such as diabetes and autoimmune diseases possibly due to their anti-inflammatory and antioxidant effects. Moreover, many studies have demonstrated the anti-cancer and cardioprotective effects of curcumin [12, 13]. In the nervous system, curcumin also shows promise for the treatment of many neurological disorders, not only for its neuroprotective properties that includes anti-protein aggregate and antioxidant activities but also by combatting glutamate excitotoxicity [14, 15]. Recently, it has also been shown to target ionotropic glutamate receptors, specifically AMPA receptors [16, 17]. In this study, the effects of the prepared curcumin derivatives on the whole cell current as well as the unique biophysical properties of AMPA subunits were investigated.

The role of GluA2 is immensely significant due to its selective permeability against divalent cations, predominantly Ca^2+^ [18]. Unlike, all other AMPAR subunits and NMDARs, GluA2 undergoes RNA editing of a single amino acid alteration (Q/R site), which regulates both the electrophysiological and ion-permeation properties of heteromeric AMPA receptors [19]. Moreover, the most abundant form of AMPA subunit is GluA2 reaching up to 45% of all AMPA subunits in the CNS [20]. Very low abundance or even absence of GluA2 containing AMPA receptors in motor neurons has been associated with ALS disease due to increased Ca^2+^ permeability, which increases the risk of excitotoxicity upon AMPAR activation. Additionally, the regulation, insertion, and expression of GluA2 subunit containing AMPARs play a unique role in various neurological disorders, that are all linked to excessive ion permeability and or activity [19, 21, 22]. Thus, the current study aims to investigate the effect of curcumin derivatives precisely on GluA2 subunits, both in the homomeric (GluA2) and heteromeric GluA2/GluA3 form of AMPA receptors.

## Materials and methods

### Curcumin derivatives

**Fig 1:**
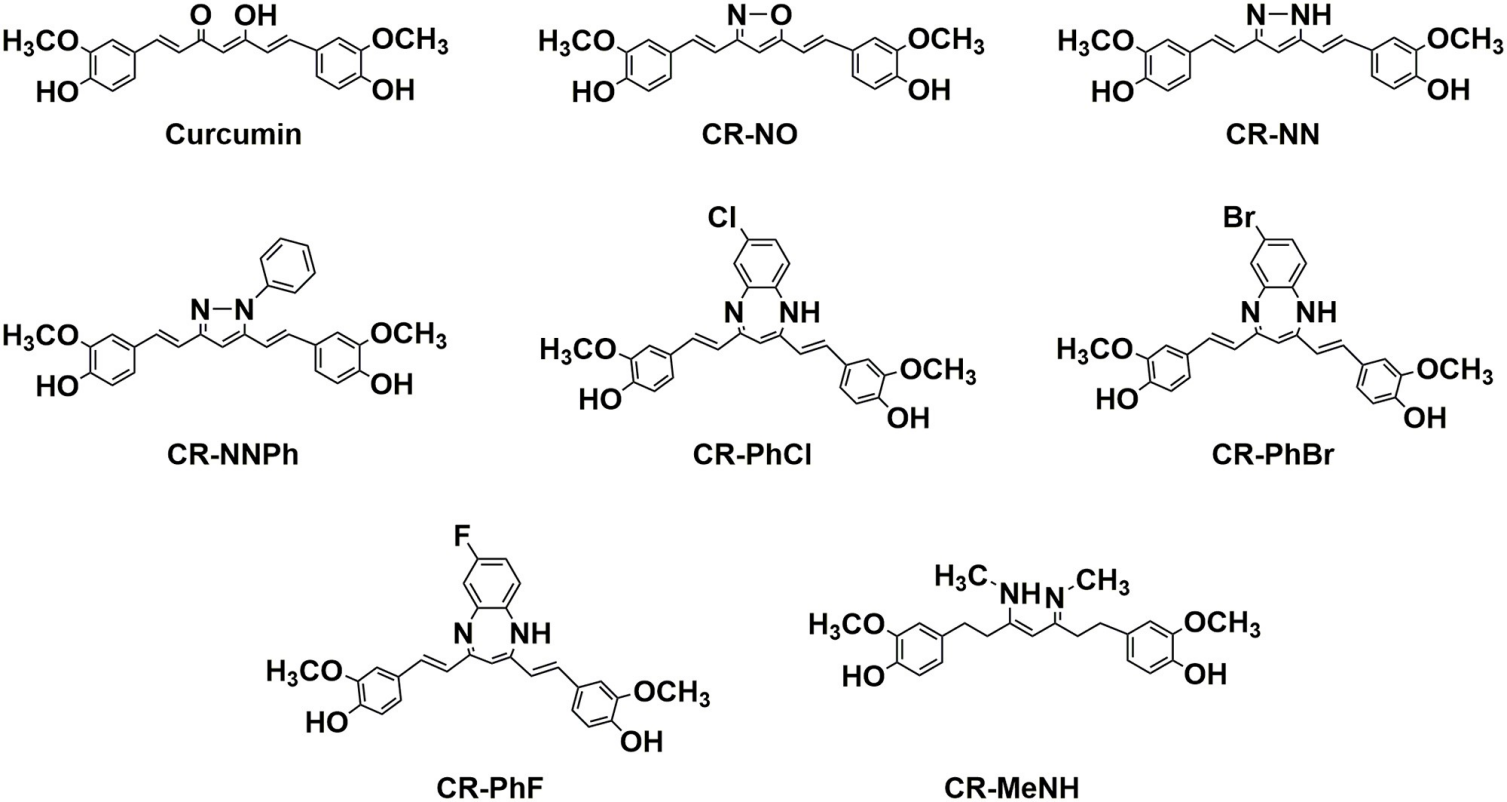
The chemical structures of the synthesized curcumin derivatives.

The synthesis of the curcumin derivatives is provided in the experimental section of the supporting information. The Curcumin and its derived drugs with different active sites (as shown in Fig 1) are the following:

4,4’-((1E,1’E)-isoxazole-3,5-diylbis(ethene-2,1-diyl))bis(2-methoxyphenol), 4,4’-((1E,1’E)-(1H-pyrazole-3,5-diyl)bis(ethene-2,1-diyl))bis(2-methoxyphenol), 4,4’-((3Z,5E)-3-(methylamino)-5-(methylimino)hept-3-ene-1,7-diyl)bis(2-methoxyphenol), 4,4’-((1E,1’E)-(7-chloro-1H-benzo[b][1,4]diazepine-2,4-diyl)bis(ethene-2,1-diyl))bis(2methoxyphenol), 4,4’-((1E,1’E)-(7-fluoro-1H-benzo[b][1,4]diazepine-2,4-diyl)bis(ethene-2,1-diyl))bis(2methoxyphenol), 4,4’-((1E,1’E)-(7-bromo-1H-benzo[b][1,4]diazepine-2,4-diyl)bis(ethene-2,1-iyl))bis(2methoxyphenol), and 4,4’-((1E,1’E)-(1-phenyl-1H-pyrazole-3,5-diyl)bis(ethene-2,1-diyl))bis(2methoxyphenol), for the sake of simplicity, the derivatives were abbreviated as; (CR-NO), (CR-NN), (CR-MeNH), (CR-PhCL), (CR-PhF), (CR-PhBr), and (CR-NNPh), respectively.

### DNA preparation

QIAGEN Plasmid Mini Kit was used to prepare up to 20 μg of high-copy plasmid DNA. A selective plate was streaked followed by the selection of a single colony. For a starter culture, the medium that was used was LB, which was inoculated containing the appropriate selective antibiotic. Afterward, the culture was incubated approximately 8 h at 37°C, which was later diluted with 3 ml selective LB medium. The culture then placed in the incubator at 37°C for roughly 12–16 h. Centrifugation was done followed by the resuspension of the formed pellet in order to harvest the bacterial cells. After the addition of 0.3 ml of Buffer P2, the sealed tube was inverted 4–6 times for homogeneity. Later, the tubes were centrifuged to obtain the supernatant containing the plasmid DNA. 1 ml Buffer QBT was used to equilibrate a QIAGEN-tip 20, the column could empty by gravity flow then the supernatant was applied to the QIAGEN-tip 20 and by gravity flow, it entered the resin. Buffer QC was used to wash the QIAGEN-tip. This was followed with the elution of the DNA with 0.8 ml buffer QF, and thenfor precipitation purposes isopropanol was also added. It was mixed and centrifuged immediately to carefully decant supernatant. Ethanol was used to wash the DNA pellet, then centrifuged again, and the supernatant was carefully removed as to not disturb the pellet. Finally, the pellet was air-dried, and the DNA was re-dissolved in a suitable volume of buffer. Running spectrophotometry at 260 nm, the quantitative analysis on agarose gel was used to calculate DNA concentration so to determine the yield. A260 readings should lie between the values of 0.1 and 1.0 to judge the reliability of spectrophotometric DNA quantification.

### cDNA transient transfection in HEK293 cells

All AMPAR subunits used in this study contained the flip isoform. GluA2 (flip isoform) in pBlueScript that was obtained from S. F. Heinemann (Salk Institute, La Jolla, CA) and sub-cloned in pRK for expression in Human Embryonic Kidney cells 293 (HEK293). (The HEK293 cell line was obtained from Sigma, Germany). The GluA2 unedited form (R607Q) (flip isoforms) and enhanced green fluorescent protein (EGFP) in pRK5 were a gift from P. H. Seeburg (Max Planck Institute for Medical Research, Heidelberg, Germany). GluA2 homomer plasmids were cotransfected with a GFP expression vector (1 μg of GluA2, 1 μg GFP) in HEK293 cells by chemical-mediated transfection. Likewise, GluA2/3 plasmids with the of ratio 1:1.2 heteromers were also transfected in the same manner. Cells were then seeded in Petri dishes in DMEM supplemented with 10% fetal calf serum and antibiotics and maintained in a humidified incubator at 37 °C and 5% CO_2_. Highly fluorescent cells were identified and selected for recording.

### HEK293 cell culture and transfection

HEK293 were grown in Dulbecco Modified Eagle Medium (DMEM) (Sigma, Germany) containing 10% FBS (fetal bovine serum), 0.1 mg/ml streptomycin, and 1 mM sodium pyruvate (Biological Industries; Beit-Haemek, Israel). HEK293 cells were incubated at 37° C and 5% CO_2_ was supplemented to the medium. It was subcultured twice a week until cells reached pass #20. The transfection reagent used was either jetPRIME (Polyplus: New York, NY) or Lipofectamine 2000 (Invitrogen; San Diego, CA). Cells were kept for 36 hrs. after transfection in 12-well plates. then replated on coverslips coated with Laminin (1 mg/mL; Sigma, Germany) to use for electrophysiology recordings.

### HEK293 cell patch-clamp recordings

Using IPA (Integrated Patch Amplifier) (Sutter Instruments, Novato, CA) on the whole cell configuration of the patch-clamp technique, HEK293 Cells were recorded 36–48 hours after transfection, at a temperature of 22°C, the membrane potential of -60 mV. SutterPatch Software v. 1.1.1 (Sutter Instruments) to digitize membrane currents for a short period. Sampling frequency was set to 10 kHz, and the low-pass filter was set to 2 kHz. Borosilicate glass was used to fabricate the patch electrodes with a resistance of 2–4 MΩ. The extracellular solution contained (values are in mM): 150 NaCl, 2.8 KCl, 0.5 MgCl_2_, 2 CaCl_2_, 10 HEPES adjusted to pH 7.4 with NaOH. The pipette solution contains (values are in mM): 110 CsF, 30 CsCl, 4 NaCl, 0.5 CaCl_2_, 10 Trypsin EDTA solution B (0.25%), EDTA (0.05%), 10 HEPES, adjusted to pH 7.2 with CsOH. Using double barrel glass (theta tube) glutamate and solutions of choice were rapidly administered, the theta tube was mounted on a high-speed piezo solution switcher (Automate Scientific, Berkeley, CA). After expelling the patch from the electrode to estimate the speed of solution exchange, the open tip potentials were recorded during the application of solutions of different ionic strengths. The 10%–90% solution exchange was typically at 500 ms. The second barrel was supplying the cells of glutamate and the derivatives individually after obtaining the current of each application. The exchange of the solution in the different tubes were interchangeable on a single cell to calculate for inhibition. Hence, inhibition was calculated by comparing the current observed by supplying the cell with glutamate alone, and with the current given after supplying the same cell with glutamate and the antagonist of interest. To ensure the safety of the cell and validate the results of the antagonist, the cell was then resupplied with the first tube containing only glutamate, which to consider viable should be almost identical to the glutamate-induced current before the application of antagonist. 6 viable cells were considered for the sample size to obtain the average inhibition by the derivative of interest. The exact data analysis of this process is provided in the supporting information. Data acquired were analyzed using Igor Pro7 (Wave Metrics, inc). Receptor desensitization (τ_des_) and deactivation rates were estimated by a single exponential fitting of the current decay starting from 95% of the peak to the baseline current. The currents were evoked by the application of 3 mM glutamate for desensitization and 1 ms of glutamate for 500 ms for deactivation. AMPAR-current deactivation and desensitization were fitted with two exponentials and the weighted tau (τ_w_) was calculated as τ_w_ = (τf x af) + (τs x as), where af and as are the relative amplitudes of the fast (τf) and slow (τs) exponential component. See supporting information for more information regarding data analysis.

## Results

### Inhibition and effect of CR-MeNH, CR-NO, CR-NN, and CR-NNPh on the biophysical gating properties of GluA2 and GluA2/A3 AMPA receptors

To observe the effect of CR-MeNH and CR-NO on the whole-cell current amplitude (A), desensitization and deactivation, HEK293 cells were recorded 36–48 hours after transfection in the absence and presence of these derivatives. To ensure an open channel state, we used a 10 mM ligand concentration at which ∼95% corresponds to the open-channel form [23]. The concentration of the antagonist was fixated at 20 μM due to lack of difference in inhibition after this point and observed level of precipitation. Regardless of glutamate concentration, no change in the inhibition nor the biophysical properties were observed as shown in the supporting information S1 Fig.

**Fig 2:**
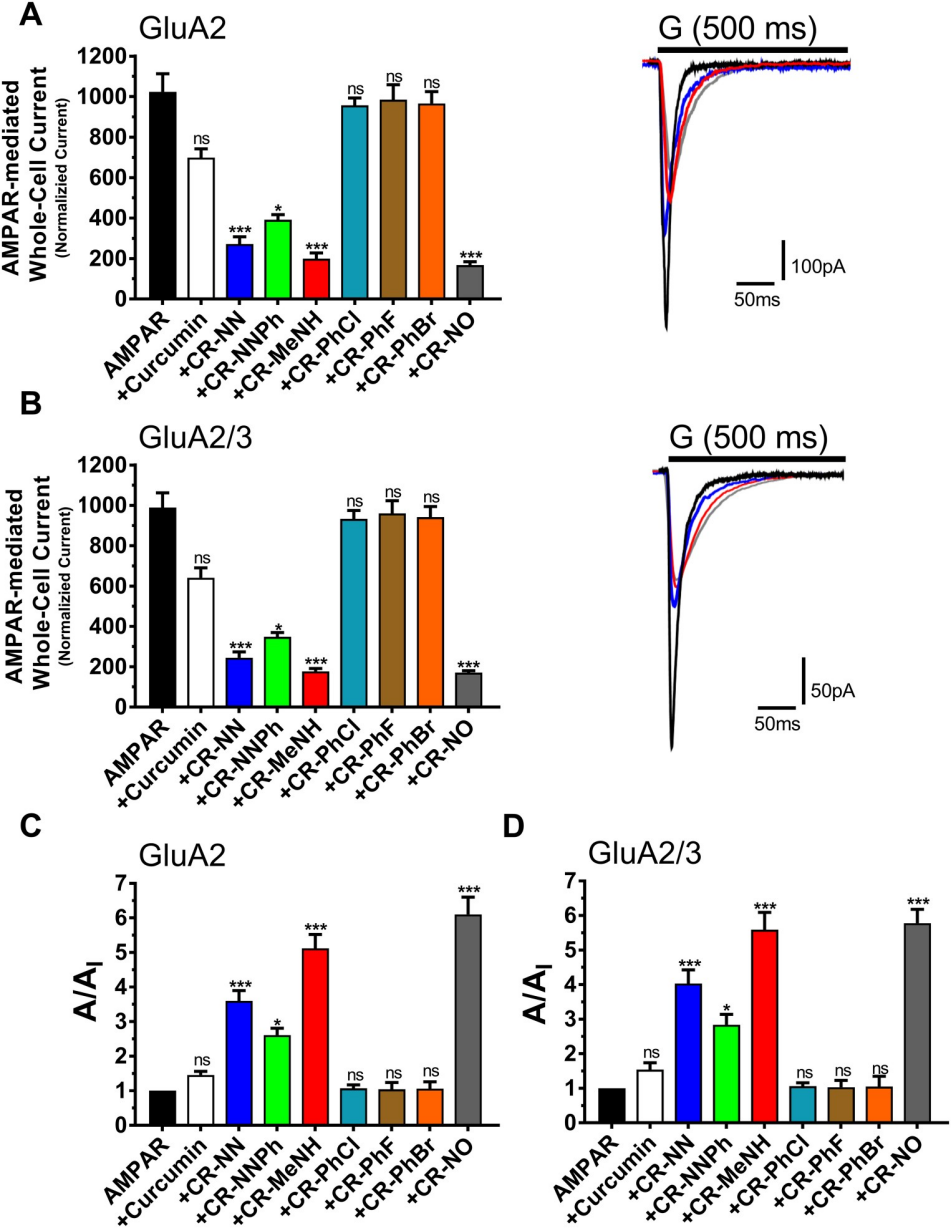
Effect of curcumin and the derivatives on the amplitude of the whole-cell current in the absence and presence of derivatives. **A** are the AMPAR recorded upon 500 ms application of 10 mM glutamate to whole-cell recording from HEK293 cells expressing homomeric GluA2 alone or in combination with the derivatives. **B** are the AMPAR recorded upon 500 ms application of 10 mM glutamate to whole-cell recording from HEK293 cells expressing heteromeric GluA2/3 alone or in combination with the derivatives. **C**-**D** Inhibition assays of different derivatives on GluA2 and GluA2/3. The whole-cell current recording was conducted at −60 mV, pH 7.4, and 22 °C. Graphs summarize weighted time constants for activation. Data shown are mean ± SEM; n = 6 (number of patch cells in the whole-cell configuration). Significance (one-way ANOVA): * *p* < 0.05; ** *p* < 0.01; *** *p* < 0.001; ns, not significant.

**Fig 3:**
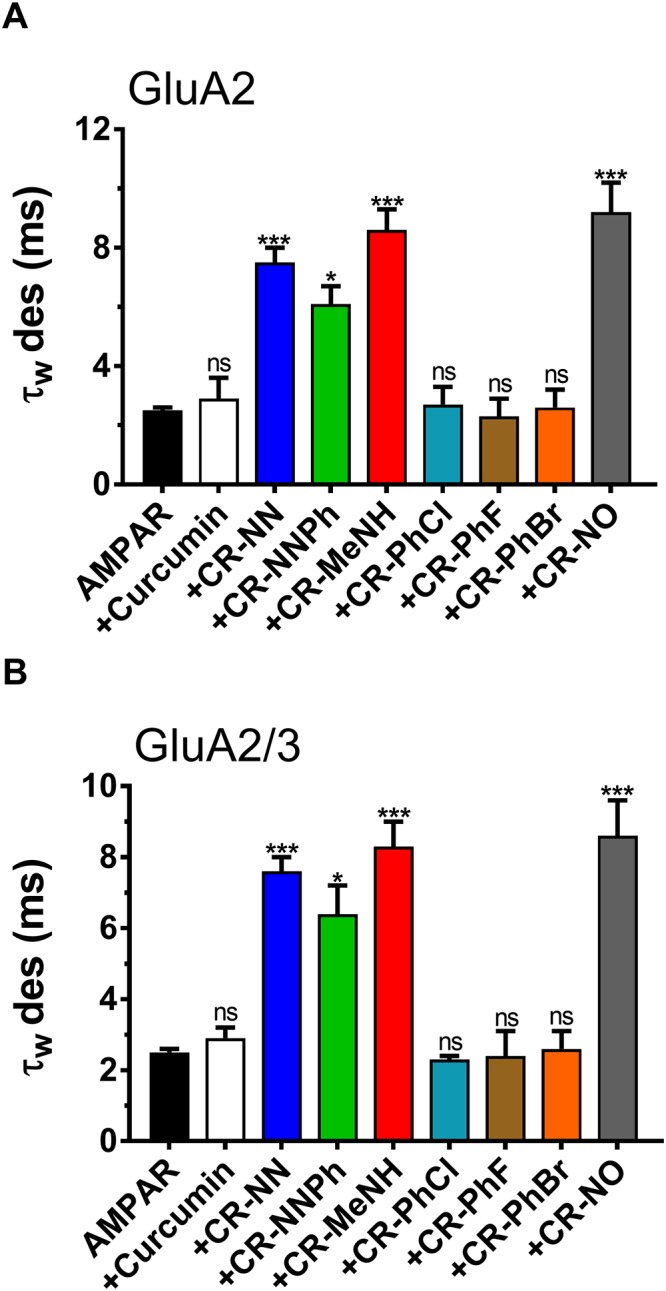
Effect of Curcumin and the derivatives on AMPAR desensitization. **A** is the desensitization time in milliseconds (ms) from HEK293 cells expressing homomeric GluA2 alone or in combination with derivatives. **B** is the desensitization time in milliseconds (ms) from HEK293 cells expressing heteromeric GluA2/3 alone or in combination with derivatives. The whole-cell current recording was conducted at −60 mV, pH 7.4, and 22 °C. Graphs summarize weighted time constants for desensitization (τ_W_ des). Data shown are mean ± SEM; n = 6 (number of patch cells in the whole-cell configuration). Significance (one-way ANOVA): * *p* < 0.05; ** *p* < 0.01; *** *p* < 0.001; ns, not significant.

**Fig 4:**
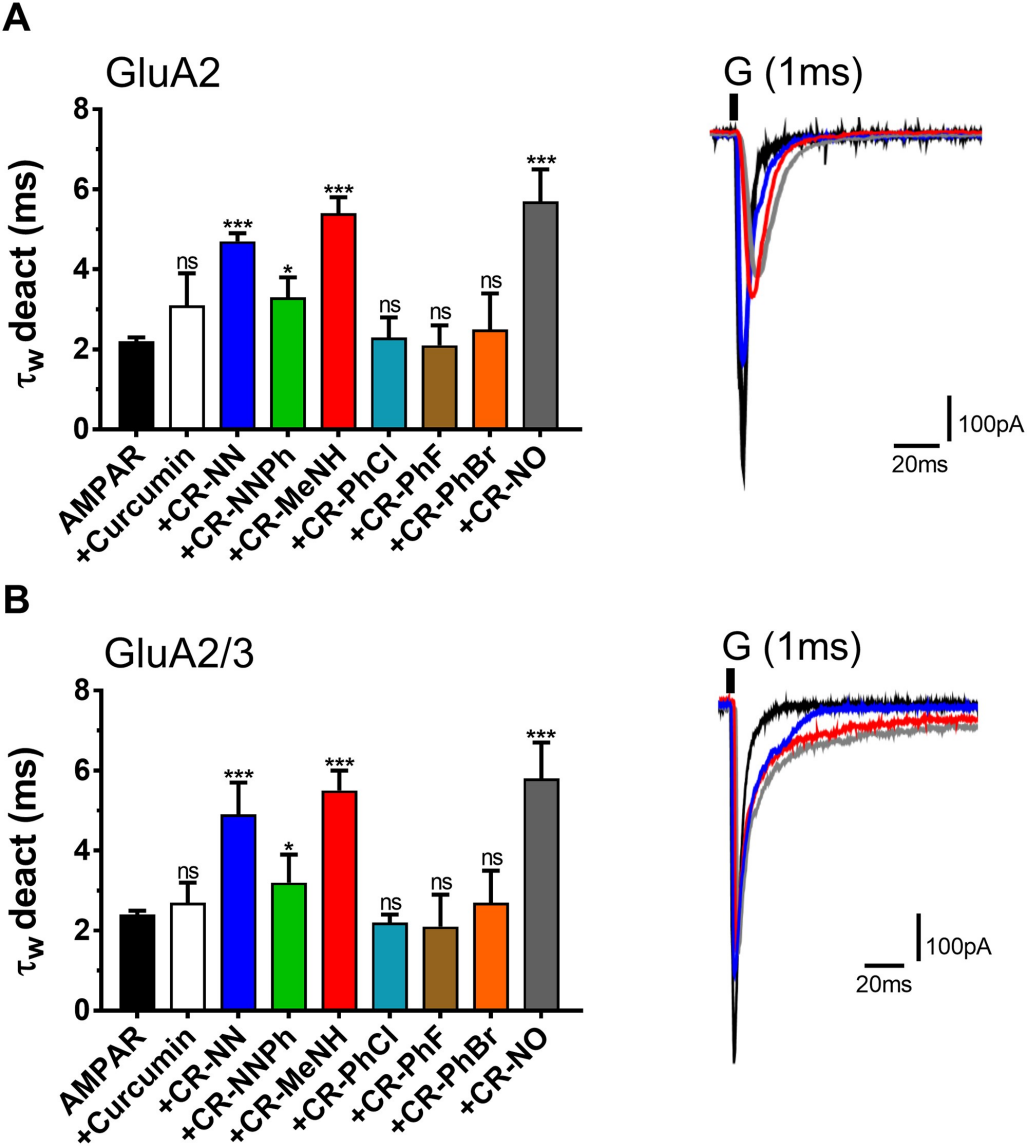
Effect of Curcumin and the derivatives on AMPAR deactivation. **A** is the deactivation time in milliseconds (ms) from HEK293 cells expressing homomeric GluA2 alone or in combination with derivatives. **B** is the deactivation time in milliseconds (ms) from HEK293 cells expressing heteromeric GluA2/3 alone or in combination with derivatives. The whole-cell current recording was conducted at −60 mV, pH 7.4, and 22 °C. Graphs summarize weighted time constants for desensitization (τ_W_ des). Data shown are mean ± SEM; n = 6 (number of patch cells in the whole-cell configuration). Significance (one-way ANOVA): * *p* < 0.05; ** *p* < 0.01; *** *p* < 0.001; ns, not significant.

Fig 2 is a representation of the amplitude and the ratio of the whole-cell current amplitude in the absence and presence of an inhibitor (i.e. A/A_I_) as a function of inhibitor concentration, where A/A_I_ = 1 represents no inhibition, Fig 3 entails the desensitization rate, and Fig 4 encompasses the deactivation rate of AMPARs subunits with and without the derivatives. Hence at a fixed concentration of 20 μM, the two most effective derivatives, CR-MeNH and CR-NO, impacted both the homomeric and heteromeric AMPA receptors roughly 5–6 folds. Testing the homomeric GluA2 for the peak current, desensitization and deactivation, without the administration of any curcumin derivative resulted with the following recordings; 1024±89 pA, 2.5±0.1 ms, and 2.2±0.1 ms. However, upon the application of CR-MeNH, the amplitude read at 200±28 pA, decreasing the current by 5.12 folds as shown in Fig 2, while increasing desensitization and deactivation to 8.6±0.7 ms and 5.4±0.4 ms, respectively. Likewise, the derivative CR-NO had a similar effect by decreasing the peak current 6.1 folds at a reading of 168±17 pA, as well as increasing desensitization to 9.2±1.0 ms as shown in Fig 3, while for the deactivation increasing it to 5.7±0.8 ms that is evident in Fig 4.

The effect of the derivatives on the heteromeric AMPA receptors GluA2/A3 had similar results. The peak current decreased 5.59 folds with CR-MeNH measuring at 177±14 pA, which was of similar recording observed by the administration of CR-NO; 171±9.0 pA (Fig 2). As for desensitization, both CR-MeNH and CR-NO had increased this state to 8.3±0.7 ms and 8.6±1.0 ms respectively (Fig 3). Finally, CR-MeNH and CR-NO effected the deactivation as follows; 5.5±0.5 ms, and 5.8±0.9 ms (Fig 4).

CR-NN derivative had a slightly higher impact than CR-NNPh on the biophysical properties and inhibition of all AMPARs. At a significance level of p = 0.01, CR-NN measured peak current (Fig 2), desensitization (Fig 3), and deactivation (Fig 4) for GluA2 homomer to equal to 272±36 pA, 7.5±0.5 ms, and 4.7±0.2 ms respectively. Similar results were obtained for heteromeric GluA2/A3 receptors under the influence of the same compound, which were as follows; 245±29 pA, 4.9±0.8 ms, and 7.6±0.4 ms. As for CR-NNPh, the derivative exhibited an impact on all AMPARs at a significant level of p = 0.05. Hence, the peak current decreased by 2.61 folds for the GluA2 homomer measuring at 392±25 pA, while for the heteromeric GluA2/A3 at 348±22 pA resulting in a decrease by 2.84 folds (Fig 2). As for the desensitization and deactivation, CR-NNPh increased both readings for the GluA2 homomer to 6.1±0.6 ms and 3.3±0.5 ms, likewise for the heteromeric GluA2/A3 the readings were increased to 6.4±0.8 ms and 3.2±0.7 ms respectively.

### The influence of CR-PhCl, CR-PhF and CR-PhBr curcumin derivatives on AMPAR desensitization, deactivation and peak current

The results obtained from the previously mentioned derivatives were not observed in the following derivatives CR-PhCl, CR-PhF, and CR-PhBr. In fact, these derivatives had no significant impact on any of the biophysical gating properties tested or showed any inhibition for either the GluA2 homomer as well as the heteromeric GluA2/A3. The administration of CR-PhCl, CR-PhF, and CR-PhBr on GluA2 homomer measured the desensitization at 2.7±0.6 ms, 2.3±0.6 ms, and 2.6±0.6 ms, while on heteromeric GluA2/A3 measurements were equal to 2.3±0.1 ms, 2.4±0.7 ms and 2.6±0.5 ms respectively (Fig 3). For the deactivation rate, the administration of these three derivatives on the GluA2 homomer resulted in the following readings; 2.3±0.5, 2.1±0.5 and 2.5±0.9, which were of similar results to the heteromeric GluA2/A3 reading at 2.2±0.2, 2.1±0.8, and 2.7±0.8, respectively (Fig 4). Finally, CR-PhCl, CR-PhF, and CR-PhBr had no significant changes on the peak current on any of the AMPARs whatsoever. The peak current for GluA2 homomer upon derivative administration measured at 957±36, 985±74 and 966±59 in respect to the prior mentioned derivatives. Likewise, heteromeric GluA2/A3 receptor’s peak current with the derivatives read as the following; 933±42, 960±63, and 942±52 (Fig 2). In comparison to its derivatives, curcumin possessed no significant impact on the biophysical gating properties of any tested AMPARs, nor showed any inhibitory actions. The readings of peak current with curcumin for GluA2 homomer was at 699±43.

## Discussion

The excitotoxicity of AMPARs has been well established in the pathology of many neurological diseases. As AMPARs are over-activated, rapid or delayed neurotoxicity is triggered, which is potent enough to cause neuronal death and neurodegeneration on a larger scale [21, 22, 24, 25]. Many experimental drugs have been designed to inhibit the activity of AMPARs in attempt to reduce neurotoxicity and to treat numerous diseases. For example, a phase four clinical trial drug is known as talampanel used to treat epilepsy, fails due to neurotoxicity and low efficacy, as it had a much weaker inhibition against AMPA than what is observed from the results of the current study [26, 27]. Moreover, while it may have been safe at a clinical II trial it was shown to be inefficacious for ALS due to its selectivity towards AMPAR subunits [28]. The only commercial drug that is currently available for AMPAR inhibition and used as an anti-epileptic is perampanel, shown to be efficacious on all AMPAR subunits [29], which is similar to the synthesized derivatives in that matter [30, 31]. Nonetheless, Perampanel users suffer from various side effects such as depression, aggression, fatigue, etc. [32]. Hence, the current study investigates the effect of derivatives synthesized from the natural polyphenol, curcumin, on the biophysical properties of AMPARs and detect any form of inhibition on the activity of the tested receptors.

This study aims to synthesize a more potent drug with a higher specificity, solubility, and efficacy. The prepared curcumin derivatives were associated with various heterocyclic moieties; pyrazole, isoxazole, and diazepine, which were also linked to a Schiff base. The purpose of using heterocyclic compounds is to increase th solubility in hydrophilic solvents and interactions with bioactive sites due to the extensive hydrogen bonds [33]. The incorporation of the Schiff base was to enhance the drugs binding affinity and potency by adding functionality that acts as a H-donor and acceptor to replace the carbonyl group in Curcumin. Several pyrazol derivatives showed a good binding affinity for AMPA receptor [34]. Also, compounds with isoxazole moiety showed high potency and selectivity towards AMPA antagonists [35]. Finally, unlike another drug candidate, these derivatives are easily synthesized at a high quantitative yield.

For both GluA2 homomer and GluA2/A3 heteromeric, the CR-MeNH and CR-NO derivatives showed the most significant inhibitory effect, by reducing the peak current up to 6 folds as shown in Fig 2b. They also reduced both deactivation and desensitization rates remarkably, denoting the property to slow the kinetics of those receptors. Likewise, CR-NN and CR-NNPh had a similar impact on both receptors, by decreasing the peak current and increasing the duration of the state at which the receptor is desensitized (Fig 3) or deactivated (Fig 4), suggesting a common mechanism shared by these derivatives to inhibit AMPARS. However, Curcumin, CR-PhCl, CR-PhF, and CR-PhBr did not show significant changes in the peak current, deactivation or desensitization.

The activation of the glutamate receptor is dependent on glutamate binding to at least one of the subunits of the receptor; thus, the more glutamate binds to subunits the more significant the activation. The effect of CR-NN, CR-NNPh, CR-MeNH and CR-NO curcumin derivatives on the peak current of AMPARS reveal a decrease in activation, insinuating that the active sites of these compounds have an affinity to an inhibitory binding site on the receptor. An increase in glutamate concentration had no observable effect on derivative activity on AMPARs, meaning they act as noncompetitive inhibitors, (see supporting information S1 Fig). For the four most potent inhibitors the ratio of inhibition, A/A_I_ was consistent regardless of glutamate concentration of 2, 4, 6, 8, 10, and 12 mM. Furthermore, allosteric sites can be targets for pharmacological agents to modulate the function of the receptor either positively or negatively [36, 37]. The mechanism of impact by negative modulators remains obscure, although some electrophysiological data indicate that negative modulators act by weakly influencing agonist binding and or alter the conformational stability of the 3D structure, a clear pathway is limited as different negative modulators bind to different sites and result in different outcomes [1, 25].

Conformational changes in the domains of an AMPAR directly control the kinetics and in turn the function of AMPARs. Hence, the activation of the receptor is due to rotational changes between dimers upon agonist binding mediated through the extracellular globular domains D1 and D2. The action of such agonists depends on binding to the S1 and S2 domains that modulate the conformational changes of the receptor and transduce it to the transmembrane domains acting as linkers between transmembrane domains and ligand binding domain [38, 39]. Three linker types have been identified; S1-M1, S2-M3, and S2-M4 linkers. Various research of 3D structures and sequence mutation on AMPARs demonstrated the location of non-competitive inhibitors to occur in the linker regions [40].

The same principle also applies to the desensitization mechanism. Conformational changes in the dimer interface mediate the termination of ion flow through the receptor despite the binding of the agonist. Continuous activation of the receptor leads to a separation in the linker domains, which is coupled with a conformational strain that requires a stable dimer interface to maintain. During desensitization, the dimer interface rearranges to a more stable conformation without transmitting a strain to a channel gate [41, 42]. Hence, desensitization occurs on a much faster timescale than activation. These biophysical properties of AMPARs are pharmacologically targeted to modulate the function of the receptors. Positive modulators block desensitization by binding to a linker region that upon activation and separation of the linkers, the dimer interface becomes increasingly stable and does not require rearrangements. Since the tested derivatives inhibited activity and decreased desensitization and deactivation rates of AMPARs we suggest the same logic underlying its noncompetitive inhibition. Hence, they can implement their effect by reversing the mechanism seen by a positive modulator. As a result, they block the separation of the linkers and thus inhibits activation but also obstruct any conformational transduction to the TMD, instead conform to a stable dimer conformation of the desensitized state.

The most popular and heavily researched AMPAR antagonist, 2–3 benzodiazepine has been speculated to deploy its inhibitory properties by binding to the S2-M3 linker region [24]. However, whole-cell patch experiments on this antagonist showed no influence on the rate of AMPA receptor desensitization [10]. In our study we notice that CR-NN, CR-NNPh, CR-MeNH, and CR-NO not only inhibited AMPARs but also increased desensitization and deactivation states of the receptor, it acts through a different mechanism than 2–3 benzodiazepine. Conversely, thiocyanate, which is believed to be affiliated with the linker S2-M4, is selective to AMPA subtype unlike our derivatives compounds, yet effects desensitization, deactivation and inhibits the receptors. This further signifies that different linkers are involved, at various degrees, in modulating the biophysical properties of AMPARs [36].

AMPA receptors have a critical role in the pathophysiology of many neurological disorders such as those discussed above. Depressing the activity of those receptors has therapeutic effects on those disorders and CR-NN, CR-NNPh, CR-MeNH, and CR-NO show such property that could be promising for future drug synthesis. Unlike, common non-competitive inhibitors of AMPARs, our derivatives also affected the biophysical gating properties of the receptors alongside inhibition, further enhancing its neuroprotective qualities. Although, all of the derivatives show an impact on the biophysical gating properties of AMPA receptors and possess inhibitory actions did so in varying degrees, suggesting a common mechanism of action on the receptors. Moreover, these derivatives showed no bias or selectivity to either of the tested receptors be it homomeric or tetrameric, insinuating that the mechanism is also universal between all AMPA subunits. Finally, when comparing the effect of the derivative being tested on a sub-unit vs. a heteromeric unit dependent manner, no changes in the results were observed, meaning the allosteric site for these derivatives is sub-unit dependent; hence it does not require the four subunits of a functional AMPA receptor to convey its impact. The different results achieved using the curcumin derivatives shed light on the possible effects of changing the chemical compositions and achieving a better understanding of the receptors’ binding sites affinity to such changes.

## Conclusion

To summarize the results of the current study, a number of the tested curcumin derivatives showed inhibitory properties, an increase in both desensitization and deactivation of AMPA receptors. The derivatives; CR-MeNH and CR-NO had the most significant impact on the receptors followed by CR-NN and CR-NNPh derivatives. On the other hand, CR-PhCl, CR-PhF and CR-PhBr, as well as curcumin did not affect either of the tested AMPARs. Moreover, the derivatives inhibition was independent of glutamate concentration, meaning they act as non-competitive inhibitors. We propose three different mechanisms for the observed effects of CR-MeNH, CR-NO, CR-NN, and CR-NNPh on AMPAR kinetics; first, the derivatives might affect AMPAR trafficking, reducing AMPAR density on the postsynaptic cleft. Second, they might alter the chemical structure of the pore-forming groups of the AMPAR ion channel, changing their electrochemical permeability to specific ions. Third, by acting as antagonistic modulators, they bind to allosteric sites that, in return, affect AMPAR conformation, stabilizing the desensitized and deactivation conformations and hindering activation. Hence, the chemical composition of these derivatives provides neuroprotective property against neurotoxicity caused by the excessive activation of AMPA Receptors. Further studies will be conducted to test the most active derivative against positive AMPA modulators such as cyclothiazide to determine the allosteric site of these derivative and to better understand the mechanism at which it deploys it influences on the receptor.

## Supporting information

S1 Fig Inhibition of AMPARs from Curcumin derivatives against various glutamate concentrations. The figure demonstrates the dose-dependent inhibition of AMPARs upon treating the cells with the four most potent compounds separately at varying glutamate concentrations (2–12 mM). The ration A/A_I_ for all compounds is consistent regardless of an increase of glutamate concentration suggesting these derivatives act as non-competitive inhibitors. The whole-cell current recording was conducted at −60 mV, pH 7.4, and 22 °C.
(TIF)

S1 File Synthesis of curcumin derivatives. (DOCX)

S1 Table Data analysis for the whole cell recordings. The currents (I) at the steady-state was normalized to the current obtained with agonist alone (I_0_) by comparing the current before and after the administration of the derivatives. Inhibition was calculated as a percentage of the difference in current amplitude for the pulse prior to antagonist application and the second pulse after current stabilization post-antagonist application. Significance compared with AMPAR expressed alone or with AMPAR+ Curcumin derivatives; p-value (one-way ANOVA): * < 0.05, ** < 0.01, *** < 0.001, ns–not significant.
(DOCX)
