# Supplementary figures and images for: Inhibition and assessment of the biophysical gating properties of GluA2 and GluA2/A3 AMPA receptors using curcumin derivatives
Source: PLoS One. 2019 Aug 27;14(8):e0221132. doi: 10.1371/journal.pone.0221132 (PMC6711591; doi:10.1371/journal.pone.0221132)

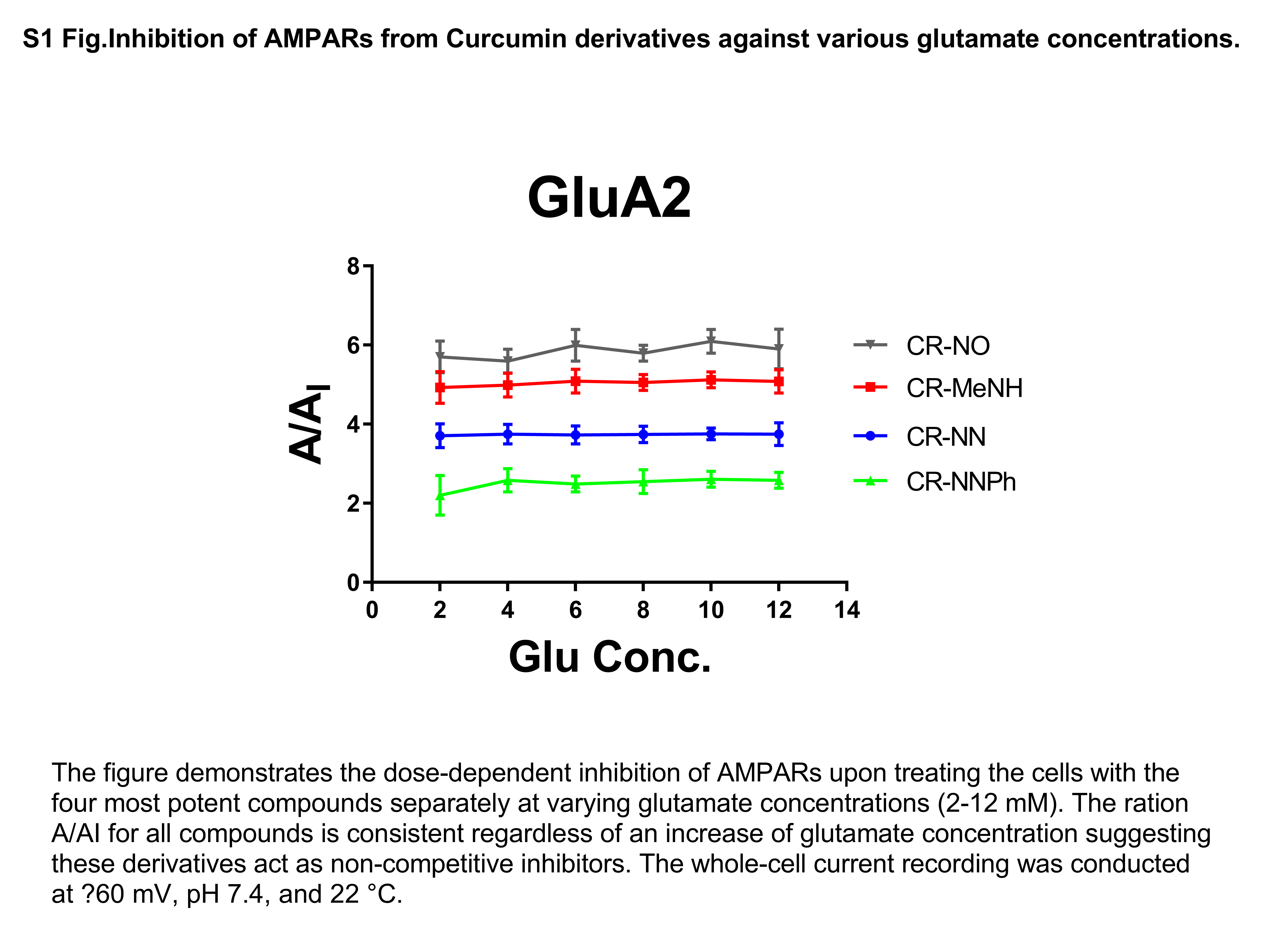

Supplement: S1 Fig — The figure demonstrates the dose-dependent inhibition of AMPARs upon treating the cells with the four most potent compounds separately at varying glutamate concentrations (2–12 mM). The ration A/AI for all compounds is consistent regardless of an increase of glutamate concentration suggesting these derivatives act as non-competitive inhibitors. The whole-cell current recording was conducted at −60 mV, pH 7.4, and 22 °C. (TIF) [file pone.0221132.s001.tif]
