# Supplementary material for: Inhibition and assessment of the biophysical gating properties of GluA2 and GluA2/A3 AMPA receptors using curcumin derivatives
Source: PLoS One. 2019 Aug 27;14(8):e0221132. doi: 10.1371/journal.pone.0221132 (PMC6711591; doi:10.1371/journal.pone.0221132)
Supplement: S1 File — (DOCX) [file pone.0221132.s002.docx]

**Inhibition and assessment of the biophysical gating properties of GluA2 and GluA2/A3 AMPA receptors using curcumin derivatives.**

Mohammad Qneibi^1,^ *, Othman Hamed^2^, Abdel-Razzak Natsheh^3^, Oswa Fares^2^, Nidal Jaradat^4^, Nour Emwas^1^, Qais AbuHasan^1^, Rana Al-Kerm^2^, Rola Al-Kerm^2^

^1^ Department of Biomedical Sciences, Faculty of Medicine and Health Sciences, An-Najah

National University, Nablus, Palestine.

^2^ Department of Chemistry, Faculty of Science, An-Najah National University, Nablus, Palestine.

^3^ Department of Computer Information Systems, Faculty of Engineering and Information

Technology, An-Najah National University, Nablus, Palestine.

^4^ Department of Pharmacy, Faculty of Medicine and Health Sciences, An-Najah National

University, Nablus, Palestine.

*Corresponding author.

E-mail address: [mqneibi@najah.edu](mailto:mqneibi@najah.edu) (MQ)

**S1 File. Synthesis of curcumin derivatives.**

All chemicals used in this study were purchased from Aldrich Chemical Company and used as they were received. All prepared compounds were characterized by MS/MS, ^1^H NMR, ^13^C NMR, IR spectroscopy. Mass spectroscopic analysis was performed on solutions of compounds in a Nano scale concentration using a multi dilution procedure. The MS/MS was performed on LCQ Fleet ion trap mass spectrometer (Thermo Fisher Scientific, CA, USA) operated in a positive electrospray mode. The electrospray voltage was 5 kV. The capillary temperature was 290°C and the sheath gas flow was 30 units. An isolation width of 2 Da was used with a 20 msec activation time for MS experiments. All scan events were acquired with a 250 ms maximum ionization time. Nuclear Magnetic Resonance spectra were recorded on Varian Gemini 2000, 300 MHz instrument MHz instruments. Infrared spectra were recorded on a Shimadzu 820 PC FT-IR spectrometer. Solvent used in the NMR was DMSO-d_6_, ^1^H NMR experiments were reported in δ units, parts per million (ppm) downfield from tetramethylsilane (TMS). All ^13^C NMR spectra were reported in ppm relative to DMSO-d_6_ (39.52 ppm). TLC analysis was performed on silica gel plates pre-coated with Merck Kiesegel 60 F254 and visualization was done using UV lamp. Samples purifications were carried out by either crystallization or by flash chromatography with silica gel (100-200) mesh.

***General experimental procedure for the preparation curcumin with 5-membered ring heterocyclic moiety***

To a round bottom flask equipped with magnetic stirring bar and a condenser, curcumin (3.0 mmole, 1.1 g) was added followed with 20.0 mL acetic acid. The desired reagent of hydrazine (3.0 mmole) or hydroxyl amine was added to the solution of curcumin. The reaction mixture was refluxed for 6 h. The reaction mixture was concentrated *in-vacuo*, and re-dissolved in ethyl acetate (50 mL). The ethyl acetate layer was washed with saturated solution of NaHCO3, saturated solution of NaCl and water. Then it was dried over Na2SO4 and concentrated *in-vacuo*. The produced solid was collected by suction filtration.

***1-phenyl-3,5-bis-2-(4-hydroxy-3-methoxystyryl)-1H-pyrazole (CR-NNPh)***

Yield 71.6% (0.96 g), mp 127-129°C, IR: *V*_max_ cm^-1^ 3550 (-C-OH), 3345 (–C–NH), 3100, 1625 (-C=N), 1610, 1585 (C=C, Ph) and 1080 (C-O ether) of (–O-CH_3_). ^1^H-NMR (300 MHz, CDCl_3_) *δ* ppm: 3.93 (s, 6H, OCH_3_), 6.07 (s, 2H, OH), 6.82 (s, 1H, C4-H), 7.05 (d, 2H, J = 14.9 Hz, C2-H and, C6-H), 7.13 (d, 2H, *J* = 14.9 Hz, C1-H and C7-H), 7.15–7.33 (m, 8H, Ph-H), 7.45 (m, 2H, Ph-H), 7.72 (m, 1H, Ph-H). ^13^C NMR (300 MHz, CDCl3) *δ* ppm: (300 MHz, CDCl3): 56.1, 56.1, 101.2, 110.0, 111.0, 112.7, 116.1, 116.2, 117.5, 120.7, 125.2, 128.2, 128.3, 128.8, 129.0, 129.1, 129.1, 131.2, 133.3, 139.7, 142.8, 147.3, 147.8, 148.1, 148.3, 151.5, 161.2. Anal. Calcd for C_27_H_24_N_2_O_4_: C 73.62, H 5.49, N 6.36. Found: C 73.43, H 5.42, N 6.48.

***4,4'-((1E,1'E)-isoxazole-3,5-diylbis(ethene-2,1-diyl))bis(2-methoxyphenol) (CR-PhNO)***

Yield 72.0 (0.8 g), mp 116-119°C, IR: *V*_max_ cm^-1^ 3560 (-C-OH), 3035, 1640 (-C=N), 1605 (C=C, 1586 (-C=C, Ph), 1330 (C-O of the five-member ring) cm-1. 1H-NMR (400 MHz, DMSO-d_6_): 1H-NMR: *δ* 3.85 (s, 6H, 2OCH3), 6.25 (s, 2H, OH), 6.71 (s, 1H, C4-H), 6.84-7.01 (m, 3H), 7.04-7.15 (m, 4H), 7.26 (m, 3H). 13C-NMR (300 MHz, CDCl_3_) *δ* ppm: 56.14, 56.18, 98.33, 110.56, 110.82, 113.12, 113.43, 115.98, 116.11, 116.24, 121.8, 122.16, 127.49, 127.83, 129.22, 135.27, 135.98, 148.22, 148.42, 162.70, 168.84. Anal. Calcd for C21H19NO5: C 69.03, H 5.24, N 3.38. Found: C 68.89, H 5.21, N 3.41.

***4,4'-((1E,1'E)-(1H-pyrazole-3,5-diyl)bis(ethene-2,1-diyl))bis(2-methoxyphenol) (CR-PhNN***)

Yield 72.0 (0.8 g), mp 116-119°C, IR: 3545 (-C-OH), 3035, 1640 (C=N), 1608, 1560 (-C=C, Ph), 1331 (C-O of the five-member ring) cm^-1^. ^1^H-NMR (400 MHz, DMSO-d_6_): 1H-NMR: *δ* 3.83 (s, 6H, 2OCH_3_), 6.25 (s, 2H, OH), 6.75 (s, 1H, C4-H), 6.81-7.07 (m, 3H), 7.07-7.16(m, 4H), 7.31 (m, 3H). ^13^C-NMR (300 MHz, CDCl_3_) *δ* ppm: 56.1, 56.1, 98.4, 110.7, 110.8, 113.1, 113.5, 115.98, 116.1, 116.2, 121.8, 122.2, 127.6, 127.8, 129.2, 135.3, 135.8, 148.2, 148.4, 162.7, 168.85. Anal. Calculated for C21H19NO5: C 69.03, H 5.24, N 3.38. Found: C 68.89, H 5.21, N 3.41.

***4,4'-((3Z,5E)-3-(methylamino)-5-(methylimino)hept-3-ene-1,7-diyl)bis(2-methoxyphenol) (CR-PhMeNH)***

Curcumin (1.0 g, 2.7 mmol) was dissolved in 50 mL of ethanol. The mixture was stirred until a clear solution was obtained. Methylamine solution 40% (0.465 g, 6 mmol) was added to the solution and the reaction mixture was refluxed for 2 h. The solvent was evaporated under vacuum, the residue was washed with 5% Na_2_CO_3_, water and dried. The residue was recrystallized from ethanol/water solution (1:2 by volume). Yield 64% (0.68 g), mp 171-175 ºC. IR: *v*_max_ cm^-1^ 3602 (-C-OH), 3050 (=C-H), 2943 (C-H), 1630 (C=N), 1610 (-C=C), 1580 (C=C, Ph), 1514, 1276, 1213, 1031. 1H-NMR (400 MHz, DMSO-d6): 1H-NMR: *δ* 2.86 (s, 6H, 2 NCH_3_)*,* 3.83 (s, 6H, 2OCH_3_), 6.27 (s, 2H, OH), 6.69 (s, 1H, C4-H), 6.79-7.05 (m, 3H), 7.06-7.19 (m, 4H), 7.28 (m, 3H). 13C-NMR (300 MHz, CDCl_3_) *δ* ppm: 31.8, 39.6, 56.24, 56.17, 98.41, 110.67, 110.93, 113.23, 113.31, 116.02, 116.32, 116.25, 121.91, 122.17, 127.52, 127.93, 129.19, 135.15, 136.06, 148.31, 148.43, 162.75, 168.8.

***General procedure for preparation of curcumin benzodiazepine derivatives***

Curcumin (**1**, 3.0 mmol) was dissolved in ethanol (30.0 mL) in a one neck round bottom flask, the mixture was stirred at room temperature until a clear solution was obtained. 1,2-diaminobenzo compound (3.0 mmol) was added to the curcumin solution followed with a 0.25 mL of concentrated sulfuric acid. The produced solution was refluxed, and the progress of the reaction was monitored by TLC. After about two hours, the reaction was complete. The solvent was removed under reduced pressure. The residue was placed on a filter paper and washed sequentially with a diluted solution of sodium carbonate (1.0%) and water. The residue was purified by recrystallization form EtOAc/hexane (40/60) solution.

***4,4'-((1E,1'E)-(7-chloro-3H-benzo[b][1,4]diazepine-2,4-diyl)bis(ethene-2,1-diyl))bis(2-methoxy phenol) (CR-PhCl)***

Bright green powder 1.31 g (96.0%), IR: *v*_max_ cm^-1^ 3530 (-C-OH), 3036, 1637 (-C=N), 1608 (C=C, aliphatic), 1582 (C=C, aromatic), 1220 (C-N), 1188 (C-O ether) and 1080 (C-O alcohol), 775 (CC-Cl). ^1^H-NMR (400 MHz, DMSO-d_6_) *δ*: 2.6 (s, 2H, CH_2_); 3.735 (s, 6H, OCH_3_), 5.45 (s, 2H, OH), 6.92-6.97 (m, 8H), 7.20 (dd, 1H, 3.4 & 12.8); 7.73 (d, 1H, J = 15.5), 7.82 (d, 1H, J = 12.7 Hz), 7.86 (d, 1H, J = 3.6 Hz). ^13^C-NMR (400 MHz, DMSO-d6) *δ*: 56.6, 112.4, 117.9, 121.1, 122.0, 125.0, 127.7, 127.7, 130.4, 132.2, 140.6, 141.9, 148.6, 150.7, 164.1. LC/MS [M + 1] for C_27_H_23_ClN_2_O_4_ Calculated 474.93, found: 475.1.

***4,4'-((1E,1'E)-(7-fluoro-3H-benzo[b][1,4]diazepine-2,4-diyl)bis(ethene-2,1-diyl))bis(2-methoxyph enol) (CR-PhF)***

Brown powder, weight 1.29 g (90.13%), IR: *v*_max_ cm^-1^ 3530 (-C-OH), 3027, 1640 (-C=N), 1607 (C=C, aliphatic), 1580 (C=C, aromatic), 1222 (C-N), 1185 (C-O ether) and 1080 (C-O alcohol), 802 (C-F). ^1^H-NMR (400 MHz, DMSO-d6) *δ*: 2.56 (s, 2H, CH_2_); 3.72 (s, 6H, OCH_3_), 5.41 (s, 2H, OH), 6.923-6.98 (m, 8H), 7.27 (dd, 1H, 3.5 & 12.1); 7.71 (d, 1H, J = 15.3), 7.85 (d, 1H, J = 12.6 Hz), 7.87 (d, 1H, J = 3.42 Hz). ^13^C-NMR (400 MHz, DMSO-d6) *δ*: 56.5, 112.4, 117.9, 121.4, 122.0, 124.1, 127.9, 127.5, 130.6, 132.2, 140.1, 142.1, 148.6, 150.7, 164.0. LC/MS [M + 1] for C_27_H_23_FN_2_O_4_ Calculated 458.18, found: 458.33.

***4,4'-((1E,1'E)-(7-bromo-3H-benzo[b][1,4]diazepine-2,4-diyl)bis(ethene-2,1-diyl))bis(2-methoxyph enol) (CR-PhBr)***

Brown solid (1.31, 90.7%). IR: *V_max_* cm^-1^ 3527(-C-OH), 3025, 1642 (-C=N), 1610 (C=C, Ph), 1584 (C=C, aromatic), 1222 (C-N), 1180 (C-O ether) and 1081 (C-O alcohol), 795 (C-Br). ^1^H-NMR (400 MHz, DMSO-d_6_) δ: 1.88 (t, 4H, CH2), 2.58 (s, 2H, CH_2_), 2.72 (t, 4H, CH_2_); 3.67 (s, 6H, OCH_3_), 5.38 (s, 2H, OH), 6.74-6.83 (m, 6H), 7.02 (dd, 1H, J = 3.4 &12.7 Hz), 7.67 (d, 1H, J = 12.7 Hz), 7.82 (d, 1H, J = 3.4 Hz). ^13^C-NMR (400 MHz, DMSO-d_6_) δ: 28.7, 34.8, 45.3, 56.40, 113.7, 116.1, 121.8, 122.1, 125.4, 127.1, 131.6, 132.9, 142.2, 144.7, 165.5. LC/MS [M + 1] for C_27_H_27_BrN_2_O_4_ Calculated 523.42, found: 523.13.
