# Supplementary material for: Inhibition and assessment of the biophysical gating properties of GluA2 and GluA2/A3 AMPA receptors using curcumin derivatives
Source: PLoS One. 2019 Aug 27;14(8):e0221132. doi: 10.1371/journal.pone.0221132 (PMC6711591; doi:10.1371/journal.pone.0221132)
Supplement: S1 Table — The currents (I) at the steady-state was normalized to the current obtained with agonist alone (I0) by comparing the current before and after the administration of the derivatives. Inhibition was calculated as a percentage of the difference in current amplitude for the pulse prior to antagonist application and the second pulse after current stabilization post-antagonist application. Significance compared with AMPAR expressed alone or with AMPAR+ Curcumin derivatives; p-value (one-way ANOVA): * < 0.05, ** < 0.01, *** < 0.001, ns–not significant. (DOCX) [file pone.0221132.s003.docx]

**Inhibition and assessment of the biophysical gating properties of GluA2 and GluA2/A3 AMPA receptors using curcumin derivatives.**

Mohammad Qneibi^1,^ *, Othman Hamed^2^, Abdel-Razzak Natsheh^3^, Oswa Fares^2^, Nidal Jaradat^4^, Nour Emwas^1^, Qais AbuHasan^1^, Rana Al-Kerm^2^, Rola Al-Kerm^2^

^1^ Department of Biomedical Sciences, Faculty of Medicine and Health Sciences, An-Najah

National University, Nablus, Palestine.

^2^ Department of Chemistry, Faculty of Science, An-Najah National University, Nablus, Palestine.

^3^ Department of Computer Information Systems, Faculty of Engineering and Information

Technology, An-Najah National University, Nablus, Palestine.

^4^ Department of Pharmacy, Faculty of Medicine and Health Sciences, An-Najah National

University, Nablus, Palestine.

*Corresponding author.

E-mail address: [mqneibi@najah.edu](mailto:mqneibi@najah.edu) (MQ)

**S1 Table. Data analysis for the whole cell recordings.**

| **Receptor Name/Compounds abbreviation** | **GluA2** | **n** | **CR-NNPh Compound** | **n** | **A/A_I_** | **CR-MeNH Compound** | **n** | **A/A_I_** |
| --- | --- | --- | --- | --- | --- | --- | --- | --- |
| **Amplitude** | 1024±89 | 6 | 392±25* | 6 | 2.61 | 200±28*** | 6 | 5.12 |
| **(pA)** |  |  |  |  |  |  |  |  |
| **τ deact** | 2.2±0.1 | 6 | 3.3±0.5* | 6 | N/R | 5.4±0.4*** | 6 | N/R |
| **(ms)** |  |  |  |  |  |  |  |  |
| **τ des** | 2.5±0.1 | 6 | 6.1±0.6* | 6 | N/R | 8.6±0.7*** | 6 | N/R |
| **(ms)** |  |  |  |  |  |  |  |  |
| **Receptor Name/Compounds abbreviation** | **GluA2/3** | **n** | **CR-NNPh Compound** | **n** | **A/A_I_** | **CR-MeNH Compound** | **n** | **A/A_I_** |
| **Amplitude** | 989±73 | 6 | 348±22* | 6 | 2.84 | 177±14*** | 6 | 5.59 |
| **(pA)** |  |  |  |  |  |  |  |  |
| **τ deact** | 2.4±0.1 | 6 | 3.2±0.7* | 6 | N/R | 5.5±0.5*** | 6 | N/R |
| **(ms)** |  |  |  |  |  |  |  |  |
| **τ des** | 2.5±0.1 | 6 | 6.4±0.8* | 6 | N/R | 8.3±0.7*** | 6 | N/R |
| **(ms)** |  |  |  |  |  |  |  |  |

| **Receptor Name/Compounds abbreviation** | **GluA2** | **n** | **CR-NNPh Compound** | **n** | **A/A_I_** | **CR-MeNH Compound** | **n** | **A/A_I_** |
| --- | --- | --- | --- | --- | --- | --- | --- | --- |
| **Amplitude** | 1024±89 | 6 | 392±25* | 6 | 2.61 | 200±28*** | 6 | 5.12 |
| **(pA)** |  |  |  |  |  |  |  |  |
| **τ deact** | 2.2±0.1 | 6 | 3.3±0.5* | 6 | N/R | 5.4±0.4*** | 6 | N/R |
| **(ms)** |  |  |  |  |  |  |  |  |
| **τ des** | 2.5±0.1 | 6 | 6.1±0.6* | 6 | N/R | 8.6±0.7*** | 6 | N/R |
| **(ms)** |  |  |  |  |  |  |  |  |
| **Receptor Name/Compounds abbreviation** | **GluA2/3** | **n** | **CR-NNPh Compound** | **n** | **A/A_I_** | **CR-MeNH Compound** | **n** | **A/A_I_** |
| **Amplitude** | 989±73 | 6 | 348±22* | 6 | 2.84 | 177±14*** | 6 | 5.59 |
| **(pA)** |  |  |  |  |  |  |  |  |
| **τ deact** | 2.4±0.1 | 6 | 3.2±0.7* | 6 | N/R | 5.5±0.5*** | 6 | N/R |
| **(ms)** |  |  |  |  |  |  |  |  |
| **τ des** | 2.5±0.1 | 6 | 6.4±0.8* | 6 | N/R | 8.3±0.7*** | 6 | N/R |
| **(ms)** |  |  |  |  |  |  |  |  |

| **Receptor Name/Compounds abbreviation** | **GluA2** | **n** | **CR-PhCl Compound** | **n** | **A/A_I_** | **CR-PhF Compound** | **n** | **A/A_I_** |
| --- | --- | --- | --- | --- | --- | --- | --- | --- |
| **Amplitude** | 1024±89 | 6 | 957±36^ns^ | 6 | 1.07 | 985±74^ns^ | 6 | 1.04 |
| **(pA)** |  |  |  |  |  |  |  |  |
| **τ deact** | 2.2±0.1 | 6 | 2.3±0.5^ns^ | 6 | N/R | 2.1±0.5^ns^ | 6 | N/R |
| **(ms)** |  |  |  |  |  |  |  |  |
| **τ des** | 2.5±0.1 | 6 | 2.7±0.6^ns^ | 6 | N/R | 2.3±0.6^ns^ | 6 | N/R |
| **(ms)** |  |  |  |  |  |  |  |  |
| **Receptor Name/Compounds abbreviation** | **GluA2/3** | **n** | **CR-PhCl Compound** | **n** | **A/A_I_** | **CR-PhF Compound** | **n** | **A/A_I_** |
| **Amplitude** | 989±73 | 6 | 933±42^ns^ | 6 | 1.06 | 960±63^ns^ | 6 | 1.03 |
| **(pA)** |  |  |  |  |  |  |  |  |
| **τ deact** | 2.4±0.1 | 6 | 2.2±0.2^ns^ | 6 | N/R | 2.1±0.8^ns^ | 6 | N/R |
| **(ms)** |  |  |  |  |  |  |  |  |
| **τ des** | 2.5±0.1 | 6 | 2.3±0.1^ns^ | 6 | N/R | 2.4±0.7^ns^ | 6 | N/R |
| **(ms)** |  |  |  |  |  |  |  |  |

| **Receptor Name/Compounds abbreviation** | **GluA2** | **n** | **CR-PhBr Compound** | **n** | **A/A_I_** | **CR-NO Compound** | **n** | **A/A_I_** |
| --- | --- | --- | --- | --- | --- | --- | --- | --- |
| **Amplitude** | 1024±89 | 6 | 966±59^ns^ | 6 | 1.06 | 168±17*** | 6 | 6.1 |
| **(pA)** |  |  |  |  |  |  |  |  |
| **τ deact** | 2.2±0.1 | 6 | 2.5±0.9^ns^ | 6 | N/R | 5.7±0.8*** | 6 | N/R |
| **(ms)** |  |  |  |  |  |  |  |  |
| **τ des** | 2.5±0.1 | 6 | 2.6±0.6^ns^ | 6 | N/R | 9.2±1.0*** | 6 | N/R |
| **(ms)** |  |  |  |  |  |  |  |  |
| **Receptor Name/Compounds abbreviation** | **GluA2/3** | **n** | **CR-PhBr Compound** | **n** | **A/A_I_** | **CR-NO Compound** | **n** | **A/A_I_** |
| **Amplitude** | 989±73 | 6 | 942±52^ns^ | 6 | 1.05 | 171±9.0*** | 6 | 5.78 |
| **(pA)** |  |  |  |  |  |  |  |  |
| **τ deact** | 2.4±0.1 | 6 | 2.7±0.8^ns^ | 6 | N/R | 5.8±0.9*** | 6 | N/R |
| **(ms)** |  |  |  |  |  |  |  |  |
| **τ des** | 2.5±0.1 | 6 | 2.6±0.5^ns^ | 6 | N/R | 8.6±1.0*** | 6 | N/R |
| **(ms)** |  |  |  |  |  |  |  |  |

The currents (I) at the steady-state was normalized to the current obtained with agonist alone (I_0_) by comparing the current before and after the administration of the derivatives. Inhibition was calculated as a percentage of the difference in current amplitude for the pulse prior to antagonist application and the second pulse after current stabilization post-antagonist application. Significance compared with AMPAR expressed alone or with AMPAR+ Curcumin derivatives; p-value (one-way ANOVA): * < 0.05, ** < 0.01, *** < 0.001, ns – not significant.
